# Bitter Taste Receptors (TAS2Rs) in Human Lung Macrophages: Receptor Expression and Inhibitory Effects of TAS2R Agonists

**DOI:** 10.3389/fphys.2019.01267

**Published:** 2019-10-02

**Authors:** Stanislas Grassin-Delyle, Hélène Salvator, Nikola Mantov, Charlotte Abrial, Marion Brollo, Christophe Faisy, Emmanuel Naline, Louis-Jean Couderc, Philippe Devillier

**Affiliations:** ^1^Department of Airway Diseases, Foch Hospital, Suresnes, France; ^2^INSERM UMR 1173, UFR Simone Veil - Santé, University Versailles Saint-Quentin, University of Paris-Saclay, Montigny-le-Bretonneux, France; ^3^Laboratory of Research in Respiratory Pharmacology–UPRES EA 220, Foch Hospital, University Versailles Saint-Quentin, University of Paris-Saclay, Suresnes, France

**Keywords:** bitter taste receptor, human lung macrophage, parenchyma explant, cytokine, inflammation

## Abstract

**Background:**

Bitter-taste receptors (TAS2Rs) are involved in airway relaxation but are also expressed in human blood leukocytes. We studied TAS2R expression and the effects of TAS2R agonists on the lipopolysaccharide (LPS)-induced cytokine release in human lung macrophages (LMs).

**Methods:**

Lung macrophages were isolated from patients undergoing surgery for carcinoma. We used RT-qPCR to measure transcripts of 16 TAS2Rs (TAS2Rs 3/4/5/7/8/9/10/14/19/20/31/38/39/43/45 and 46) in unstimulated and LPS-stimulated (10 ng.mL^–1^) LMs. The macrophages were also incubated with TAS2R agonists for 24 h. Supernatant levels of the cytokines TNF-α, CCL3, CXCL8 and IL-10 were measured using ELISAs.

**Results:**

The transcripts of all 16 TAS2Rs were detected in macrophages. The addition of LPS led to an increase in the expression of most TAS2Rs, which was significant for TAS2R7 and 38. Although the promiscuous TAS2R agonists, quinine and denatonium, inhibited the LPS-induced release of TNF-α, CCL3 and CXCL8, diphenidol was inactive. Partially selective agonists (dapsone, colchicine, strychnine, and chloroquine) and selective agonists [erythromycin (TAS2R10), phenanthroline (TAS2R5), ofloxacin (TAS2R9), and carisoprodol (TAS2R14)] also suppressed the LPS-induced cytokine release. In contrast, two other agonists [sodium cromoglycate (TAS2R20) and saccharin (TAS2R31 and 43)] were inactive. TAS2R agonists suppressed IL-10 production – suggesting that this anti-inflammatory cytokine is not involved in the inhibition of cytokine production.

**Conclusion:**

Human LMs expressed TAS2Rs. Experiments with TAS2R agonists’ suggested the involvement of TAS2Rs 3, 4, 5, 9, 10, 14, 30, 39 and 40 in the inhibition of cytokine production. TAS2Rs may constitute new drug targets in inflammatory obstructive lung disease.

## Introduction

Inflammatory lung diseases such as asthma and chronic obstructive pulmonary disease (COPD) are characterized by airway obstruction and airflow limitation. The bitter taste receptors (TAS2Rs) constitute a family of around 25 G-protein coupled receptors (GPCRs) initially thought to be located exclusively on the tongue, where their activation enables our perception of a bitter taste. However, TAS2Rs are now known to be expressed in the human bronchus (Deshpande et al., 2010; Grassin-Delyle et al., 2013), airway epithelial cells (Shah et al., 2009; Jaggupilli et al., 2017), mast cells (Ekoff et al., 2014) and blood leukocytes (Orsmark-Pietras et al., 2013; Malki et al., 2015).

In human airway smooth muscle, TAS2R stimulation induces relaxation (Deshpande et al., 2010; Grassin-Delyle et al., 2013); inhaled bitter tastants also decreased airway obstruction in a mouse model of asthma (Deshpande et al., 2010). The motile cilia of human airway epithelial cells express TAS2Rs, and bitter compounds increase the ciliary beat frequency as part of an airway defense mechanism (Shah et al., 2009). In sinonasal epithelial cells, activation of TAS2R38 stimulates an increase in nitric oxide production, which in turn increases mucociliary clearance and directly kills bacteria (Lee et al., 2012; Cohen, 2017). The polymorphisms that underlie TAS2R38’s functionality appear to be involved in susceptibility to upper respiratory bacterial infections (Lee et al., 2012; Cohen, 2017). In IgE-receptor–activated primary human mast cells, agonists known to bind to the expressed TAS2Rs were found to inhibit the release of histamine and prostaglandin D_2_ (Ekoff et al., 2014). Furthermore, blood leukocytes from children with severe asthma display elevated TAS2R expression levels and two TAS2R agonists inhibited the release of several pro-inflammatory cytokines and eicosanoids in whole blood from adults (Orsmark-Pietras et al., 2013). In general, TAS2R agonists may have both anti-inflammatory properties and bronchodilatory activities.

Lung macrophages are involved in the pathophysiology of pulmonary diseases like asthma or COPD, where the cells orchestrate inflammatory reactions via the release of cytokines and chemokines (Balhara and Gounni, 2012; Yang et al., 2012; Barnes, 2016). The expression of TAS2Rs in lung macrophages (LMs) has not previously been assessed. There are few old reports on the effect of certain bitter tastants (such as chloroquine, colchicine, erythromycin or ofloxacin) on cytokine release from mouse peritoneal macrophages (Ogino et al., 2009), monocyte/macrophage cell lines (Jeong and Jue, 1997; Ianaro et al., 2000; Jang et al., 2006; Schierbeck et al., 2010), human blood monocytes and monocyte-derived macrophages/dendritic cells (Yasutomi et al., 2005; Jang et al., 2006; Schierbeck et al., 2010; Vrancic et al., 2012). However, human blood monocytes or monocyte-derived macrophages are surrogate cell models that do not adequately recapitulate the biology of primary LMs (Jin et al., 2004; Jeyaseelan et al., 2005; Li et al., 2007; Groot-Kormelink et al., 2012; Tomechko et al., 2015).

The objectives of the present study were to (i) characterize TAS2R expression in human LMs, (ii) describe the inhibitory effects of various TAS2R agonists on lipopolysaccharide (LPS)-induced cytokine production, (iii) infer the subtypes of TAS2R involved, and (iv) determine whether IL-10 acts as a potential mediator of TAS2R inhibitory activities on LPS-induced cytokine production.

## Materials and Methods

### Drugs and Chemicals

The TAS2R agonists chloroquine diphosphate, quinine hydrochloride dihydrate, saccharin sodium hydrate, denatonium benzoate, 1,10-phenanthroline hydrochloride monohydrate, ofloxacin, strychnine hemisulphate, erythromycin, dapsone, carisoprodol, and sodium cromoglycate were obtained from Sigma-Aldrich (Saint-Quentin Fallavier, France) and diphenidol hydrochloride was provided by TCI Europe (Zwijndrecht, Belgium). All products were solubilized and diluted in sterile water, with the exception of erythromycin, dapsone, and carisoprodol, which were solubilized in DMSO and then diluted in water. Antibiotics, DMSO, L-glutamine, trypan blue dye, heat-inactivated fetal calf serum and LPS (from *E. coli* serotype 0111:B4) were purchased from Sigma (St. Louis, MO, United States). RPMI medium was from Eurobio Biotechnology (Les Ulis, France).

### Preparations of Human Lung Macrophages and Explants

Experiments on human tissue were approved by the regional independent ethics committee board (*Comité de Protection des Personnes Île de France VIII*, Boulogne-Billancourt, France). In line with the French legislation on clinical research and as approved by the independent ethics committee, patients gave their verbal informed consent for the use of resected lung tissue for *in vitro* experiments. Lung tissue samples were obtained from 28 patients [18 males and 10 females; smoker/ex-smoker/non-smoker: 11/16/1; mean ± standard error (SD) age: 65.4 ± 8.1 years; FEV1 = 80.2 ± 20.9%; pack-years: 41 ± 21; FEV1/FVC ratio: 0.77 ± 0.1; 7 COPD (FEV1/FVC < 0.7; airflow limitation severity: GOLD 1 for 4 patients and GOLD 2 for 3)] undergoing surgical resection for lung carcinoma and who had not received prior chemotherapy. The LMs were isolated from macroscopically normal lung parenchyma (obtained from sites distant from the tumor), dissected free of pleura, visible airways and blood vessels, and then chopped into 3–5 mm^3^ fragments, as previously described (Jeyaseelan et al., 2005; Buenestado et al., 2010, 2012; Abrial et al., 2015; Victoni et al., 2017).

Briefly, the fluid collected from several washings of the minced peripheral lung tissues was centrifuged (2000 rpm for, 10 min). The cell pellet was resuspended in RPMI supplemented with 10% heat-inactivated fetal calf serum, 2 mM L-glutamine, and antibiotics. Resuspended viable cells (10^6^ per mL) were then aliquoted into either a 12-well plate (for transcriptional assays) or a 24-well plate (for cytokine assays). Following incubation for at least 2 h at 37°C (in a 5% CO_2_ humidified atmosphere), non-adherent cells were removed by gentle washing. The remaining cells were maintained at 37°C and 5% CO_2_ overnight. It has been shown that the adherence step does not significantly influence overall transcriptional changes in alveolar macrophages, relative to flow-cytometry-based cell-sorting (Shaykhiev et al., 2009). As described in previous reports from our group (Jeyaseelan et al., 2005; Buenestado et al., 2010, 2012; Abrial et al., 2015; Victoni et al., 2017), the adherent cells (about 2 × 10^5^ cells per well, for a 24-well plate) were >95% pure macrophages, as determined by May-Grünwald-Giemsa staining and CD68 immunocytochemistry (data not shown). Cell viability exceeded 90%, as assessed in a trypan blue dye exclusion assay. Culture plates with adherent macrophages were washed with warm medium. One mL of fresh medium supplemented with 1% fetal calf serum was added per well, and the culture plates were incubated overnight at 37°C in a 5% CO_2_ humidified atmosphere.

### Treatment of Lung Macrophages

On the day after isolation, macrophages or explants were washed twice, and 1 mL of RPMI was added per well. The LMs were exposed for 24 h to LPS (10 ng^⋅^mL^–1^). The latter LPS concentration was selected as being submaximal on the basis of previous data from time-response and concentration-response curves (Buenestado et al., 2012). TAS2R agonists were added to the culture medium 1 h before exposure to LPS. After a 24 h incubation in RPMI at 37°C (in a 5% CO2 humidified atmosphere), culture supernatants from the LMs and the explants were collected and stored at −80°C for subsequent cytokine assays. Although more than 100 molecules have been described as TAS2R agonists, some TAS2Rs are still orphan receptors that lack a cognate agonist (Meyerhof et al., 2010; Di Pizio and Niv, 2015). In an initial series of experiments, the preparations were exposed to a maximal concentration (1 mM) of promiscuous TAS2R agonists acting on at least 8 different TAS2Rs (diphenidol, quinine and denatonium) (Table 1) (Meyerhof et al., 2010; Di Pizio and Niv, 2015). In a second series of experiments, the preparations were exposed to a range of concentrations of more selective TAS2R agonists (chloroquine, dapsone, strychnine, sodium cromoglycate, ofloxacin, phenanthroline, erythromycin, and carisoprodol) to infer the TAS2R subtype.

**TABLE 1:** Compounds selected for functional studies with regard to their ability to act as agonists (or not) of the 25 TAS2R receptors identified in humans to date.

***hTAS2R***	**1**	**3**	**4**	**5**	**7**	**8**	**9**	**10**	**13**	**14**	**16**	**19**	**20**	**30**	**31**	**38**	**39**	**40**	**41**	**42**	**43**	**45**	**46**	**50**	**60**
**Promiscuous agonists:**																									
Diphenidol	100		100		10 and 675 ± 186^∗^			30	30	10	100		100	100	3	100	100	30			30		30		
Quinine			10 and 838 ± 24^∗∗^		10 and 983 ± 257^∗^			10		10					10		10	10			10		10		
Denatonium			300			1000		3 and 120 ± 56	30					0.03 and 0.27 ± 0.06			100				300		30 and 240 ± 192		
**Partly selective agonists:**																									
Colchicine			100 and 1025 ± 1219														3000						300 and 1580 ± 170		
Chloroquine		10 and 172 ± 29			+/0^∗^			10000									100								
Dapsone			100					100										30							
Strychnine					+/1000^∗^			3 and 21.8 ± 7.5															0.1 and 0.43 ± 0.02		
Saccharin						+									170 ± 10 and 1700 ± 20						80 ± 60 and 1100 ± 10				
Sodium cromoglycate					3000 and 4500 ± 1600								10 and 45 ± 25								3000				
**Selective agonists:**																									
Ofloxacin							EC_50_ ≈ 200																		
Phenanthroline				100																					
Erythromycin								300																	
Carisoprodol										100															

*The most recent nomenclature was adopted (Andres-Barquin and Conte, 2004; Sainz et al., 2007; Dotson et al., 2008; Meyerhof et al., 2010; Alexander et al., 2011; Soares et al., 2013). Unless otherwise stated, single values (i.e., lacking ± SEM) represent the threshold concentration (i.e., the lowest concentration (μM) to have elicited a calcium response in transfected HEK-293T cells), whereas values presented as the mean ± SEM represent the EC_50_ (μM) (Meyerhof et al., 2010). When both a threshold concentration and the EC_50_ were available, both values were reported (in that order). When no quantitative indicators were available, the symbol “+” indicates agonism. The TAS2R numbers in bold type and those underlined correspond to the TAS2Rs found to be expressed in human LMs. The TAS2R numbers in italics correspond those not expressed in human LMs. The TAS2R numbers in standard type correspond to the receptors not assessed in RT-qPCR assays. ^∗^ values from Liu et al. (2018) and ^∗∗^ values from Jaggupilli et al. (2019).*

### Cytokine Assays

The cytokine concentrations in the supernatant (TNF-α, IL-10, CCL3, and CXCL8) were measured with an ELISA (R&D Systems), according to the manufacturer’s instructions. The assays’ limits of detection were 4 pg.mL^–1^ for CCL3, 8 pg.mL^–1^ for TNF-α, 30 pg.mL^–1^ for IL-10, and 32 pg.mL^–1^ for CXCL8. The supernatants were diluted with RPMI as appropriate, and the optical density was determined at 450 nm using a microplate reader (MRX II, Dynex Technologies, Saint-Cloud, France). Cytokine concentrations were expressed in pg.10^–6^ LMs, unless otherwise stated. Cytotoxicity was determined by measuring the lactate dehydrogenase (LDH) activity in LM supernatants (LDH assay, Cayman Chemical, Montigny-le-Bretonneux, France) after 24h exposure to the vehicle or the TAS2R agonists.

### Reverse Transcriptase – Quantitative Polymerase Chain Reaction (RT-qPCR) Analysis

RT-qPCR experiments were performed as previously described, with some modifications (Grassin-Delyle et al., 2013). Lung macrophages were stimulated (or not) for 24 h with LPS, and total RNA was prepared using TRIzol^®^ reagent (Life Technologies, Saint Aubin, France). The amount of RNA extracted was estimated by spectrophotometry at 260 nm (Biowave DNA; Biochrom, Cambridge, United Kingdom) and its quality was assessed in a microfluidic electrophoresis system (RNA Standard Sensitivity kits for Experion^®^, BioRad, Marnes-la-Coquette, France). After treatment with DNase I (Life Technologies, Saint Aubin, France), 1 μg of total RNA was subjected to reverse transcription (SuperScript^®^ III First-Strand SuperMix kit, Life Technologies). The resulting cDNA was then used for quantitative real-time PCR experiments with TaqMan^®^ chemistry (Life Technologies). The amplification was carried out using 20 ng cDNA (Gene Expression Master Mix, Life Technologies) in a StepOnePlus thermo cycler (Life Technologies). The conditions were as follows: initial denaturation at 95°C for 10 min, and then 40 cycles of annealing/extension (95°C for 15 s and then 60°C for 1 min). Fluorescence was measured at each cycle, and the real-time PCR’s threshold cycle (Ct) was defined as the point at which a fluorescence signal corresponding to the amplification of a PCR product was detected. The reaction volume was set to 10 μL. The expression of transcripts of the 16 TAS2R-encoding genes (*TAS2R3, TAS2R4, TAS2R5, TAS2R7, TAS2R8, TAS2R9, TAS2R10, TAS2R14, TAS2R19, TAS2R20, TAS2R31, TAS2R38, TAS2R39, TAS2R43 TAS2R45*, and *TAS2R46*) in the LMs was analyzed using a specific TaqMan^®^ array with predesigned reagents (Assay-on-Demand^®^, Life Technologies). In order to confirm the extraction of intact cellular mRNA and standardize the quantitative data, the reference gene coding for hypoxanthine phosphoribosyltransferase (*HPRT1*) was amplified as the same time.

### Statistical Analysis

Values in the text and figures are expressed as the mean ± standard error of the mean (SEM) unless otherwise stated from experiments with *n* independent donors. The quantitative data obtained from RT-qPCR experiments was expressed as the relative expression (2^–Δ*Ct*^), where ΔC_*t*_ is the difference between the target gene C_*t*_ and the mean C_*t*_ of the reference gene.

Data were evaluated by using either a one-way analysis of variance for repeated measures (followed by Dunnett’s *post hoc* test for multiple comparisons) or a paired Student’s t-test, as appropriate. The threshold for statistical significance was set to *p* < 0.05.

## Results

### Expression of Bitter Taste Receptor Gene Transcripts in Lung Macrophages

**FIGURE 1:**
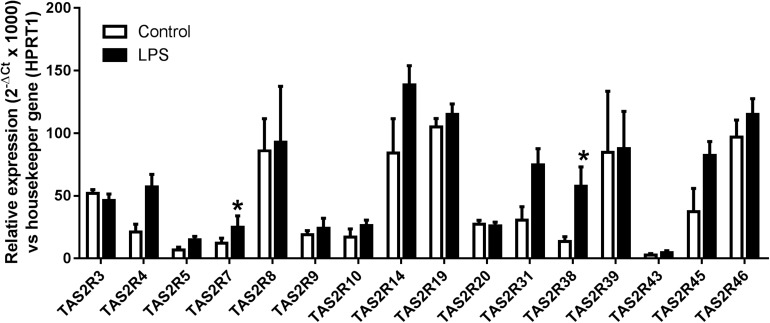
Relative expression (2^–Δ*Ct*^ × 1000) of *TAS2R3, 4, 5, 7, 8, 9, 10, 14, 19, 20, 31, 38, 39, 43, 45*, and *46* gene transcripts in human LMs (*n* = 6–12). All transcripts were found to be expressed in all patients, with the exception of *TAS2R43* (found in LMs from 4 out of 6 patients only). ^∗^*p* < 0.05 relative to the corresponding TAS2R in non-treated controls.

We analyzed the expression patterns of 16 of the 25 known TAS2Rs in humans, based on our previous results in human bronchi (Grassin-Delyle et al., 2013) and other literature data in human airway smooth muscle (Deshpande et al., 2010), airway epithelial cells (Shah et al., 2009; Jaggupilli et al., 2017) and blood leukocytes (Orsmark-Pietras et al., 2013; Malki et al., 2015). Transcripts of genes coding for 15 bitter taste receptors were identified in all the preparations (*n* = 5 to 12), whereas *TAS2R43* transcripts were found in LMs from 4 of 6 patients only. Exposure to LPS was generally associated with an increase in TAS2R receptor expression; the increase was statistically significant for *TAS2R7* and *TAS2R38* (Figure 1).

### Effects of TAS2R Agonists on Cytokine Production by Human Lung Macrophages

**FIGURE 2:**
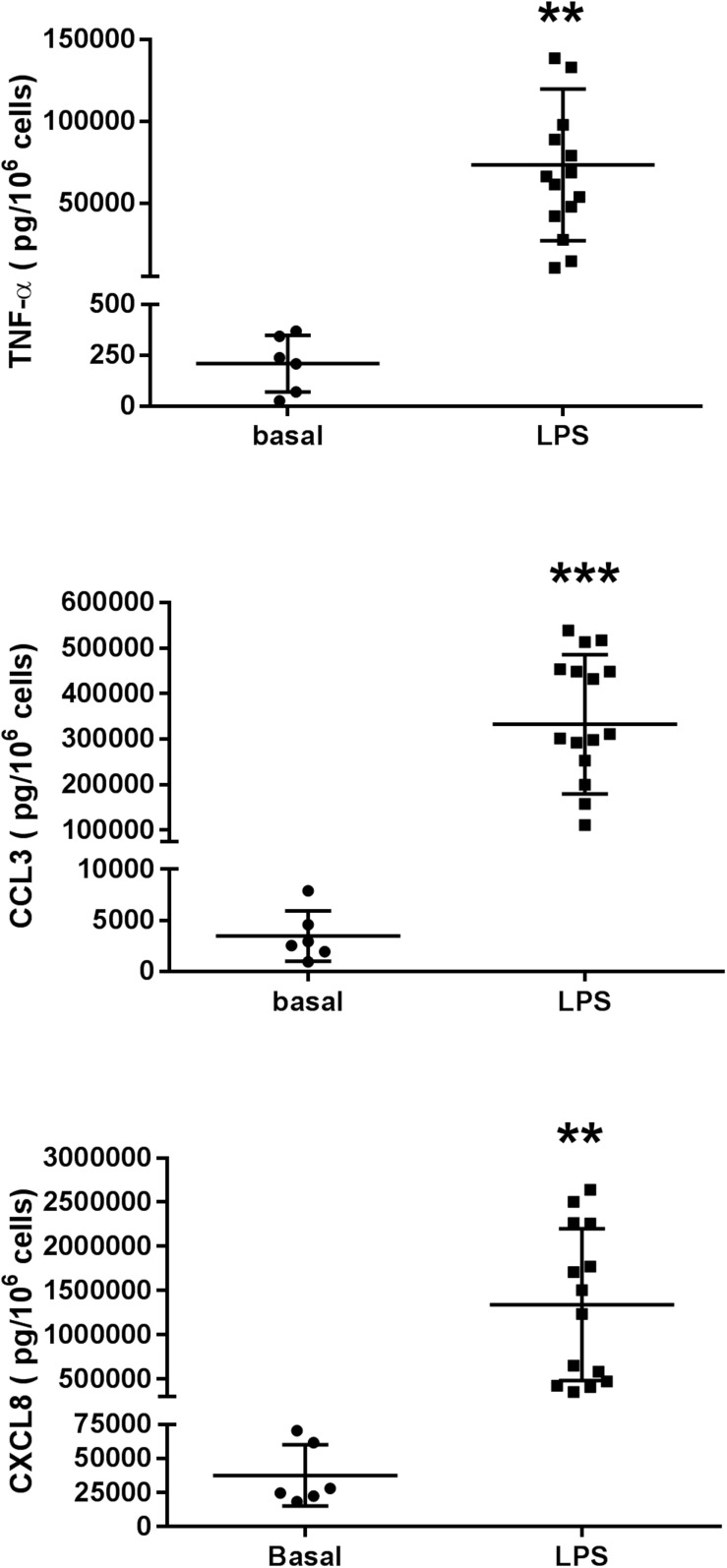
Production of TNF-α, CCL3, and CXCL8 in unstimulated human lung macrophages from 6 patients and in LPS (10 ng.mL^–1^)-stimulated human lung macrophages from 14 to 15 patients. The results are presented as individual plots and as the mean ± SD level for the group.

Lipopolysaccharide induced a ∼300-fold increase in TNF-α release (from 209 ± 56 pg.10^–6^ LMs in the absence of LPS to 63151 ± 12561 pg.10^–6^ LMs in the presence of LPS; *n* = 6 paired preparations). In the same preparations, the LPS-induced increases in chemokine release were lower: an ∼80-fold increase for CCL3 (from 3467 ± 1009 pg.10^–6^ LMs in the absence of LPS to 276235 ± 71937 pg.10^–6^ LMs in the presence of LPS) and a 34-fold increase for CXCL8 (from 37 ± 9 pg.10^–6^ LMs in the absence of LPS to 1279 ± 347 pg.10^–6^ LMs in the presence of LPS); these findings are in agreement with our previous results (Buenestado et al., 2012; Abrial et al., 2015; Victoni et al., 2017; Grassin-Delyle et al., 2018). The basal release of these cytokines in unstimulated macrophages and the release caused by LPS are shown in Figure 2.

**FIGURE 3:**
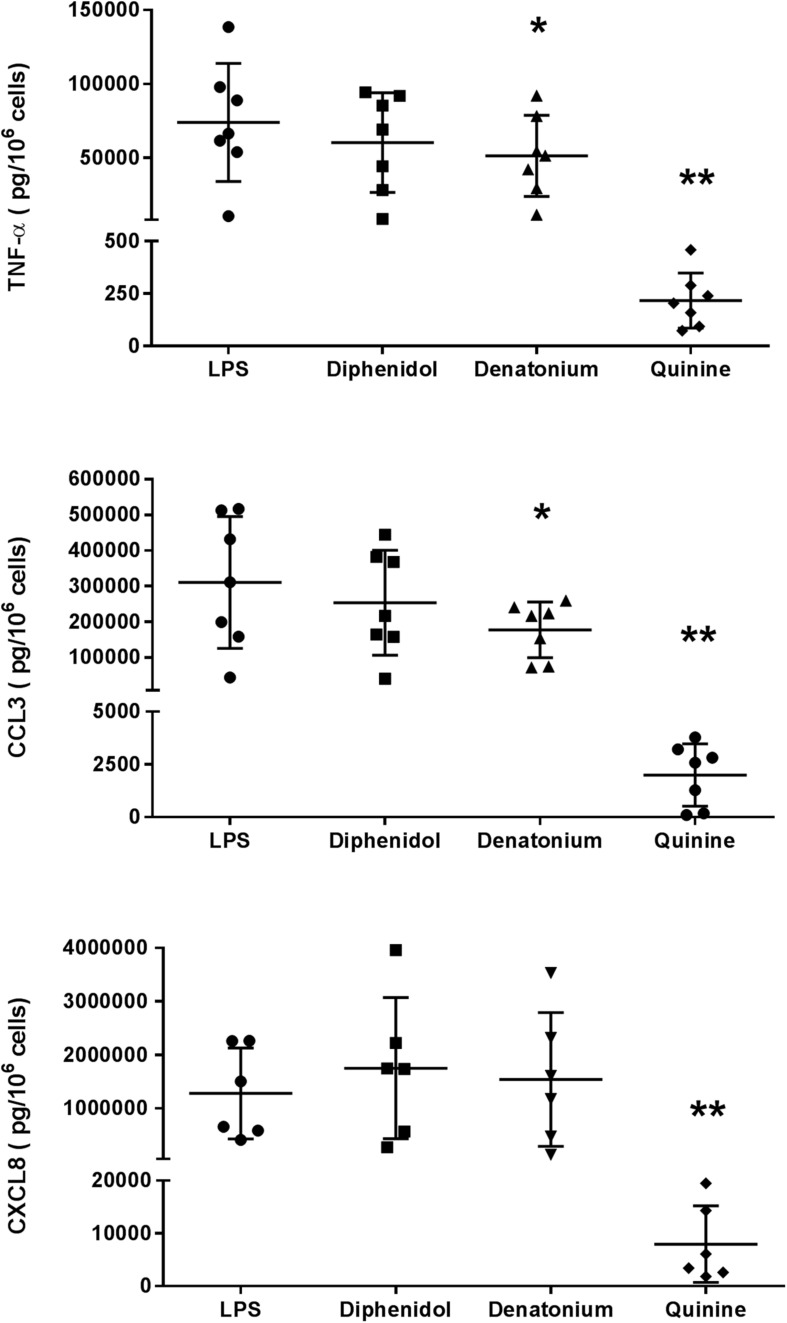
Inhibitory effects of the three promiscuous TAS2R agonists on the LPS-induced production of pro-inflammatory cytokines (TNF-α, CCL3, and CXCL8). Lung macrophages from 6 to 7 patients were stimulated with LPS (10 ng.mL^–1^) in the absence or presence of the TAS2R agonists (1 mM). ^∗^*p* < 0.05, ^∗∗^*p* < 0.01 compared with LPS alone.

The first set of experiments was carried out with three promiscuous TAS2R agonists (diphenidol, quinine, and denatonium) at a concentration of 1 mM (in order to cover the widest possible range of receptors; Table 1). Quinine almost completely abrogated the LMs’ LPS-induced production of TNF-α, CCL3 and CXCL8 (inhibition ≥98%), and denatonium significantly inhibited the production of TNF-α and CCL3 (but not of CXCL8) to a much lesser degree (28% and 43%, respectively). Diphenidol was inactive (Figure 3). Quinine is known to activate all of its nine cognate TAS2Rs in the same relatively low concentration range (∼10 μM; Table 1) (Meyerhof et al., 2010; Liu et al., 2018; Jaggupilli et al., 2019). In marked contrast, the activation ranges for denatonium and diphenidol spanned five and three orders of magnitude, respectively (Table 1) (Meyerhof et al., 2010). Given the panels of TAS2R activated by each of these three agonists and the respective activation concentrations, this first set of experiment suggested that the inhibitory effects of quinine and denatonium are almost certainly not mediated by TAS2Rs 1, 7, 13, and 31, and probably not mediated by TAS2Rs 14, 16, 20, 30, 38, 43, and 46.

**FIGURE 4:**
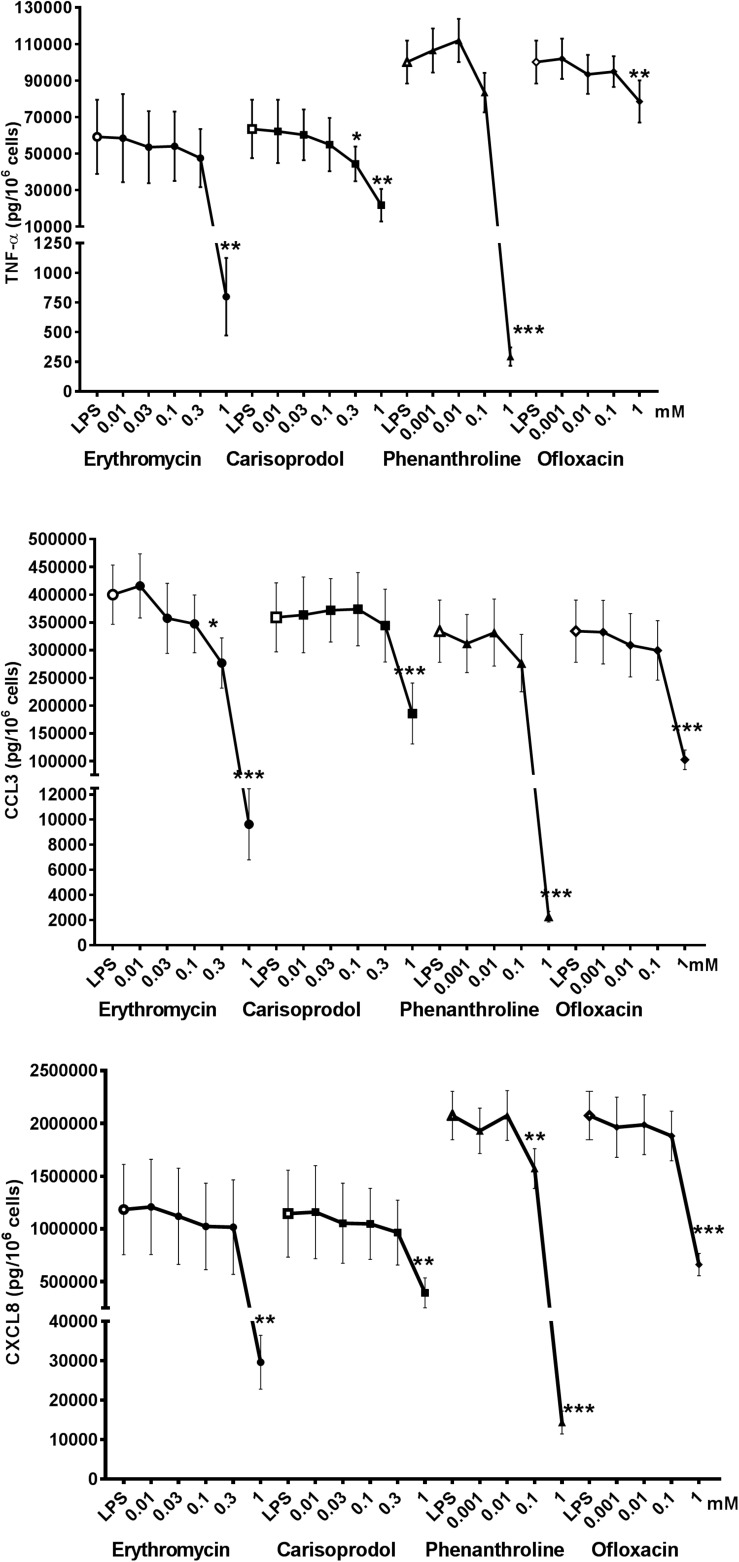
Concentration-response curves for relatively selective TAS2R agonists (colchicine, chloroquine, strychnine, and dapsone) with regard to the LPS-induced production of pro-inflammatory cytokines [TNF-α (🌑) CCL3 (■), and CXCL8 (▲)]. Lung macrophages from 5 to 9 patients were stimulated with LPS (10 ng.mL^–1^) in the absence or presence of TAS2R agonists. ^∗^*p* < 0.05, ^∗∗^*p* < 0.01, ^∗∗∗^*p* < 0.001 compared with LPS alone.

To further determine the TAS2R subtypes involved in the modulation of cytokine release by LMs, relatively selective agonists (Table 1) were used at concentrations ranging from 1 μM to 1 mM. The significant inhibitory activity of dapsone at 0.3 mM (increasing up to 1 mM) suggests the involvement of TAS2Rs 4, 10, and 40 (Figure 4).

The involvement of TAS2R4 is also indicated by the inhibitory effects of colchicine. Moreover, the observation that partial inhibition (38–57%) by strychnine was observed at a much higher concentration (1 mM) than its EC_50_ value for TAS2R46 expressed by transfected HEK-293T cells (Table 1) suggests that the latter receptor is not involved but does not rule out the involvement of TAS2R10. Chloroquine was fully active at a concentration of 10^–4^ M, suggesting the involvement of TAS2R3 and (to a lesser degree) TAS2R39.

**FIGURE 5:**
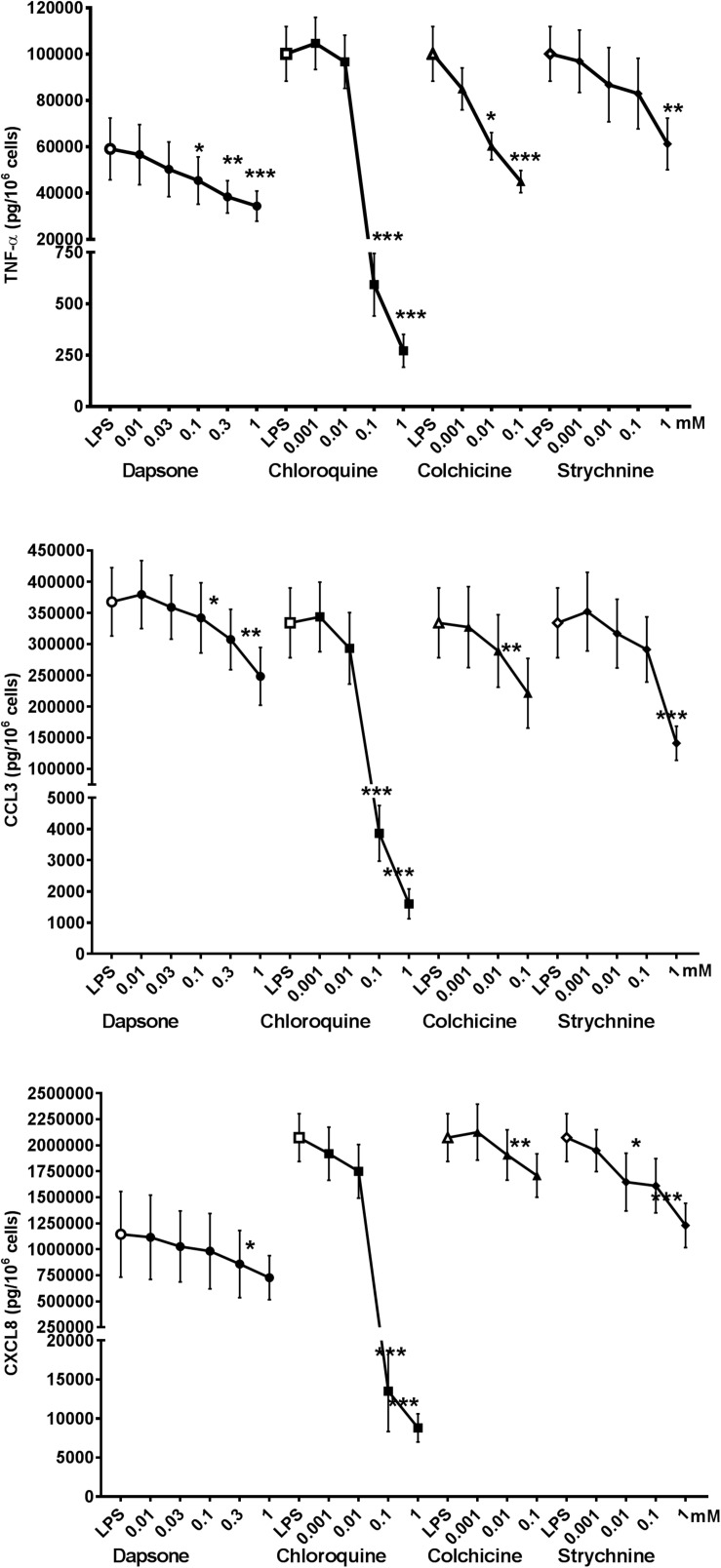
Concentration-response curves of selective TAS2R agonists [ofloxacin (TAS2R9), phenanthroline (TAS2R5), erythromycin (TAS2R10), and carisoprodol (TAS2R14)] on the LPS-induced production of pro-inflammatory cytokines [TNF-α (🌑) CCL3 (■), and CXCL8 (▲)]. Lung macrophages from 5 to 8 patients were stimulated with LPS (10 ng.mL^–1^), in the absence or presence of TAS2R agonists. ^∗^*p* < 0.05, ^∗∗^*p* < 0.01, ^∗∗∗^*p* < 0.001 compared with LPS alone.

Erythromycin, phenanthroline, ofloxacin, and carisoprodol are considered to be selective for a single (different) subtype of TAS2R. Erythromycin is a relatively weak agonist at 1 mM (activation threshold concentration: 0.3 mM) at TAS2R10, although off-target activities could also explain the compound’s inhibitory activity; solid evidence of the involvement of this subtype is hard to obtain. Phenanthroline is a selective TAS2R5 agonist; indeed, it is the only TAS2R5 agonist to have been described to date (Meyerhof et al., 2010). The compound’s potent inhibitory effect in the present study suggests the involvement of this TAS2R subtype. Ofloxacin is selective for TAS2R9, and was associated with significant inhibition of LPS-induced CCL3 and CXCL8 release, which also suggests the involvement of this TAS2R subtype. Carisoprodol is reportedly selective for TAS2R14, with an activation threshold concentration of 0.1 mM. Its significant inhibition of LPS-induced release of the three cytokines suggests the involvement of the TAS2R14, and is consistent with quinine’s inhibitory profile here (Figure 5).

Lastly, sodium cromoglycate (a selective TAS2R20 agonist, at an active concentration of 1 mM) and saccharin (an active agonist of both TAS2R31 and 43 at 1 mM) were devoid of activity (data not shown; *n* = 7 and 8, respectively); this finding rules out the involvement of these three TAS2R subtypes, as had already been indicated by our results with the promiscuous agonists. None of the TAS2R agonists caused a significant increase in LDH release, with the exception of a ∼2-fold-increase in LDH release for chloroquine and colchicine at their highest concentrations (1 mM, and 0.1 mM); this corresponds to small increases (1.6 and 1.9%, respectively) above the level of basal LDH release by LMs exposed to LPS.

Overall, cross-tabulation of the effects on LPS-induced cytokine release suggests the highly probable involvement of TAS2Rs 3, 4, 5, 9, 10, 30, 39 and 40 and (with a degree of inconsistency) TAS2R14. The high level of TAS2R14 expression further supports the involvement of this subtype. In contrast, the involvement of TAS2Rs 20, 31 and 43 can be ruled out. Lastly, the results obtained with the present set of agonists could not preclude the involvement of other TAS2Rs (particularly, the orphan receptors TAS2Rs 19, 42, and 50). It is noteworthy that the maximal level of inhibition (≥90%) observed with quinine, chloroquine, phenanthroline and erythromycin was similar to that seen for budesonide at 10^–8^ M (Table 2).

**TABLE 2:** Inhibitory effect of budesonide on the LPS-induced production of cytokines.

**Cytokine (ng/10^6^ LMs)**	**LPS**	**LPS + Bud 10 nM**	**Inhibition (%)**
TNF-α *n* = 5	64.8 ± 9.4	8.2 ± 2.3 ^∗∗^	88.2 ± 3.0
CCL3 *n* = 5	277.3 ± 29.1	22.1 ± 5.9 ^∗∗∗^	92.2 ± 2.1
CXCL8 *n* = 5	1851.7 ± 209.2	78.3 ± 13.1 ^∗∗∗^	95.8 ± 1.2
IL-10 *n* = 5	3.2 ± 2.0	0.16 ± 0.06 ^∗∗∗^	95.2 ± 1.0

*Human LMs were incubated for 1 h in the presence of budesonide (10 nM) or vehicle before being stimulated with LPS (10 ng.mL^–1^) for 24 h. The concentrations of the cytokines in the culture supernatant were measured using ELISAs. The data are reported as the mean ± SEM of five independent paired experiments. ^∗^ indicates a significant difference relative to LPS alone (^∗∗^*p* < 0.01 and ^∗∗∗^*p* < 0.001).*

### Effects of TAS2R Agonists on IL-10 Production by Human Lung Macrophages

**FIGURE 6:**
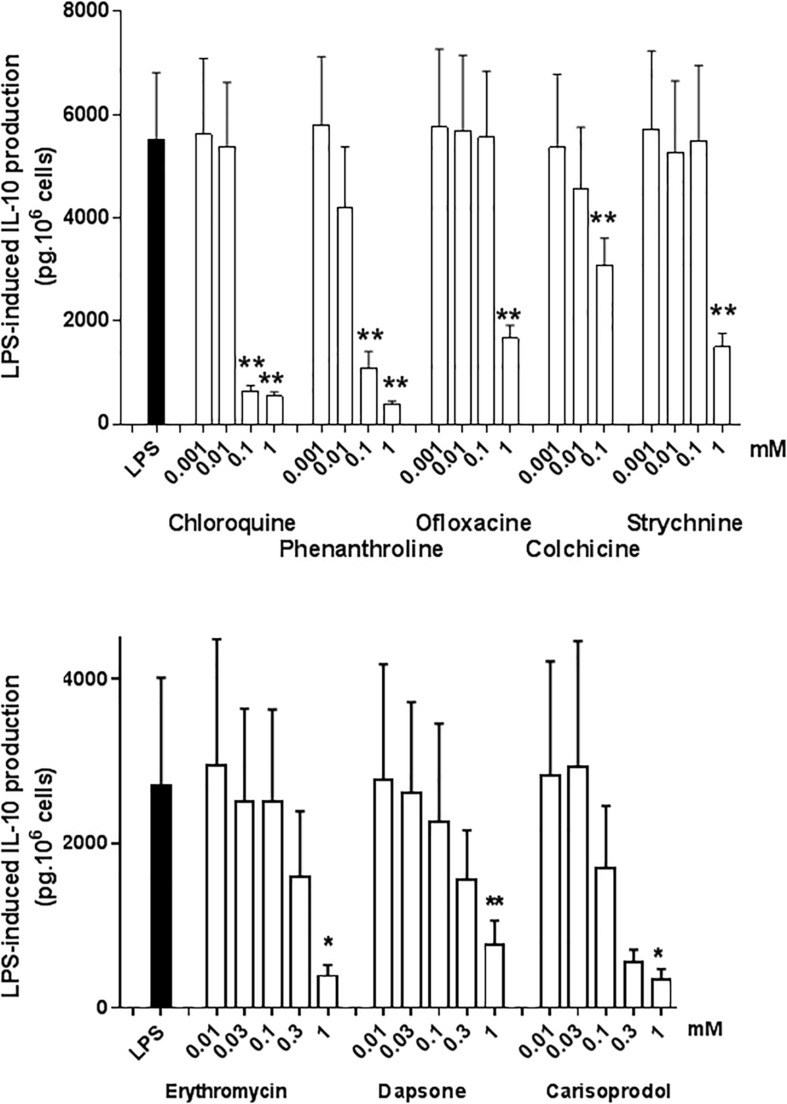
Concentration-response of TAS2R agonists on the LPS-induced production of IL-10. Lung macrophages from 4 to 5 patients were stimulated with LPS (10 ng.mL^–1^) in the absence or presence of TAS2R agonists. ^∗^*p* < 0.05, ^∗∗^*p* < 0.01.

Interleukin-10 is an immunomodulatory cytokine with potent anti-inflammatory activity; as such, it may be an essential mediator of the TAS2R agonists’ ability to inhibit the LPS-induced production of pro-inflammatory cytokines (Berkman et al., 1995; Armstrong et al., 1996). All the TAS2R agonists that inhibited the production of TNF-α, CCL3 and CXCL8 also inhibited (to much the same degree) the LPS-induced production of IL-10. The compounds that did not inhibit the LPS-induced production of TNF-α, CCL3 and CXCL8 did not alter IL-10 production either (Figure 6). Budesonide (10^–8^ M) also markedly reduced IL-10 production (by 95%; Table 2).

## Discussion

Our present results demonstrated that (i) TAS2Rs are indeed expressed in human LMs, and (ii) TAS2R agonists inhibit LPS-induced cytokine release by LMs – a process that is not mediated by the release of IL-10.

Eleven TAS2Rs (TAS2Rs 4, 5, 10, 13, 14, 19, 20, 31, 45, 46, and 50) were expressed in blood leukocytes from children, and some were upregulated in blood leukocytes from patients with severe, treatment-resistant asthma. Overall, TAS2R expression levels are higher in T lymphocytes than in monocytes in blood from adult patients with asthma (Orsmark-Pietras et al., 2013). The relatively weak expression of TAS2Rs in monocytes versus lymphocytes (i.e., B, T and natural killer cells) has been confirmed in healthy adults (Malki et al., 2015). The most abundant TAS2R transcripts in human monocytes differed in the two previously mentioned literature studies. Overall, the greatest expression levels were seen for TAS2Rs 14, 31, 41, 43, 45 and 46. Our study is the first to have demonstrated the expression of sixteen different TAS2Rs in human LMs. The most abundant transcripts in unstimulated LMs were TAS2Rs 8, 14, 19, 39 and 46, and TAS2Rs 31, 38 and 45 after exposure to LPS – highlighting differences in expression patterns between human monocytes and LMs. Since almost all the lung tissue donors were smokers or ex-smokers, we cannot rule out the possibility that smoking had altered the expression of TAS2Rs by LMs. The present work was limited by the fact that it did not investigate TAS2R expression at the protein level (using flow cytometry or Western blotting, for example). However, due the very limited availability of suitable antibodies, studies of the cell surface expression of TAS2R on human blood monocytes or monocyte-derived macrophages have been mainly restricted to TAS2R38 and TAS2R43/31 (Malki et al., 2015; Maurer et al., 2015; Gaida et al., 2016; Tran et al., 2018). However, our present results suggest that these two TAS2Rs are not involved in the inhibitory effects of the TAS2R agonists on the LPS-induced production of cytokines.

There are very few specific antagonists for use as chemical probes of TAS2R function. It has been reported that one antagonist (GIV3727) fully suppressed the activation of six TAS2Rs. GIV3727 resembles bitter agonists in that it acts on several receptors (Behrens and Meyerhof, 2013). Some 6-methoxyflavanones have been described as reversible, insurmountable antagonists of TAS2R39 (Roland et al., 2014). However, this TAS2R subtype is unlikely to be involved in inhibition of the LPS-induced production of cytokines by LMs. Given the current lack of appropriate specific TAS2R antagonists, we sought to infer the receptor subtypes involved in the inhibitory effect of the bitter taste compounds by cross-tabulation of the results for the effect of various TAS2R agonists on LPS-induced cytokine production. In Meyerhof et al.’s (2010) extensive work on HEK cells expressing the different hTAS2Rs, the researchers described the molecular receptive ranges of the 25 human TAS2Rs vs. 104 natural or synthetic bitter compounds. The “threshold concentrations” (defined as the minimum concentrations that elicit a response) and the potencies (EC_50_) determined for some compounds using calcium imaging analysis of transfected HEK cells may not be transposable to the LMs. However, Meyerhof et al.’s (2010) pioneering work was the basis for our choice of the different TAS2R agonists used in the present study (Table 1). In the human LM model, our cross-tabulation of the TAS2R agonists’ effects on LPS-induced cytokine release suggests that TAS2Rs 3, 4, 5, 9, 10, 14, 30, 39 and 40 (but not 20, 31, and 43) were involved. In a recent publication (Jaggupilli et al., 2019), levofloxacine (the S (-) enantiomer of ofloxacin) was shown to activate TAS2R4, TAS2R14 and TAS2R20 in transfected HEK293T cells further supporting the involvement of TAS2R4 and TAS2R14.

One of the strengths of the present study is its use of a broad panel of TAS2R agonists. One of the limitations relates to potential non-TAS2R-mediated (off-target) activities of some of the agonists, which might have interfered with our analysis and interpretation of the results. For example, the lysosomotropic compound chloroquine inhibits lysosomal functions and also reportedly causes a concentration-dependent suppression of the LPS-induced release of TNF-α, IL-1β, and IL-6 in human monocytes and in monocyte/macrophage cell lines (U937, THP-1, and RAW 264.7) (Jang et al., 2006; Schierbeck et al., 2010). The decrease in TNF-α release has been attributed to chloroquine’s ability to block the conversion of pro-TNF-α to mature TNF-α, whereas the decrease in IL-1β and IL-6 release has been attributed to destabilization the corresponding mRNAs (Jang et al., 2006; Schierbeck et al., 2010). Furthermore, chloroquine reportedly degrades *in vitro* cell viability at concentrations above 100 μM (Jeong and Jue, 1997; Jang et al., 2006; Schierbeck et al., 2010). However, in addition to the very weak increase in the LDH release by chloroquine (present study), the inhibition of LPS-induced cytokine production was significant at 100 μM in the previous studies (Jeong and Jue, 1997; Jang et al., 2006; Schierbeck et al., 2010) and was substantially complete in the present study – thus ruling out a cytotoxic effect. Macrolides have much the same cationic and lysosomal properties as chloroquine. In the murine monocyte/macrophage cell line J774, four macrolide antibiotics (including erythromycin and azithromycin) reduced the LPS-stimulated production of TNF-α, IL-1β, and IL-6 in a concentration-dependent manner (up to 80 μM) (Ianaro et al., 2000). The impairment of lysosomal functions by azithromycin and chloroquine deregulates TLR4 recycling/signaling and phospholipase activation – leading to an anti-inflammatory phenotype in LPS-stimulated J774 cells (Munic et al., 2011; Nujic et al., 2012). In human monocytes, azithromycin (50 μM) precisely mirrored the effects of chloroquine on LPS-induced cytokine production, with lower levels of some cytokines (CCL22 and CXCL11), higher levels of others (CCL2 and CCL18), and no changes in TNF-α and IL-6 levels (Vrancic et al., 2012). The effects of azithromycin and chloroquine may not be entirely due to impairments in lysosomal functions or in signaling pathways related to NF-κB activation, cellular accumulation and phospholipid binding (Vrancic et al., 2012). To the best of our knowledge, only one study has shown that certain macrolides (clarithromycin and azithromycin, but not erythromycin) can inhibit cytokine production by human alveolar macrophages; clarithromycin was more effective than azithromycin at ∼10 μM (Cai et al., 2013). In the present study, erythromycin suppressed LPS-induced cytokine production (TNF-α, CCL3, and CXCL8) at a ∼10-fold higher concentration in LMs than in J774 cells. This effect is probably related (at least in part) to activation of TAS2R10 (Meyerhof et al., 2010).

In monocytes and macrophages, cytoskeletal microtubules are involved in a number of cell activities, including cytokine production. Relative to microtubules in monocytes, alveolar macrophage microtubules are longer, more numerous and much more stable, and LPS increases the number and stability of monocyte microtubules still further (Allen et al., 1997). These differences might explain why treatment with 25 μM colchicine (a microtubule-depolymerizing drug) gave a relative increase in LPS-induced IL-1β release and a relative decrease in LPS-induced TNF-α release by human monocytes but had much weaker effects on human alveolar macrophages (Allen et al., 1991). In the present study, however, colchicine was associated with concentration-dependent inhibition of the production of TNF-α and CCL3; this inhibition was relatively weak at 10 μM but significant at 100 μM. These concentrations suggest that colchicine’s effect is exerted (at least in part) through TAS2R receptor activation.

In human blood monocytes, murine monocyte cell lines, and bone marrow-derived dendritic cells stimulated with LPS, chloroquine and azithromycin significantly enhance IL-10 release (Sugiyama et al., 2007; Murphy et al., 2008; Vrancic et al., 2012). Since IL-10 has broad anti-inflammatory properties (Berkman et al., 1995; Armstrong et al., 1996), we assessed the effects of TAS2R agonists on the production of this cytokine by LMs. TAS2R agonists that inhibited the production of TNF-α, CCL3, and CXCL8 also inhibited IL-10 production to a similar extent. Budesonide also markedly reduced the IL-10 production. Hence, in contrast to the situation in monocytes, IL-10 is not involved in the TAS2R agonists’ inhibitory effects in LMs. The molecular mechanisms underlying the anti-inflammatory effects of TAS2R activation in human lung macrophages should be investigated as soon as selective, potent TAS2R agonists and antagonists become available. Taken as a whole, these results suggest that a battery of selective TAS2R agonists and antagonists will be needed to confirm our findings and then to establish the full list of receptors involved in the inhibition of LPS-induced cytokine release. With respect to drug efficacy, some of the active TAS2R agonists (quinine, chloroquine, phenanthroline, and erythromycin) led to the essentially complete inhibition of LPS-induced cytokine production by LMs (to much the same extent as an optimal concentration of budesonide), which illustrates the potent anti-inflammatory activity of these compounds and the potential therapeutic value of inhaled TAS2R agonists in obstructive pulmonary diseases. Interestingly, it has been reported that chloroquine has beneficial effects in asthma (Charous et al., 1998). Given the potential TAS2R-mediated effects observed here, it is time to review the actions of chloroquine and related compounds in airway diseases.

Another potential study limitation relates to our use of LMs harvested from lung tissues resected from current smokers or ex-smokers. Isolation from minced lung tissue provides the large number of cells required to perform paired series of experiments, such as those described here. This work would be hardly possible with macrophages obtained from bronchoalveolar lavages. It should be noted that our preparations might contain a small proportion of interstitial resident macrophages among the alveolar macrophages. However, exposure to bacterial products (including LPS) and inhaled TAS2R agonists is not restricted to the alveolar compartment, and the use of freshly isolated human LMs mainly from the alveolar spaces but perhaps also from the lung tissue might usefully reflect the clinical response more closely.

About a quarter of the lung tissue donors studied here had COPD and almost all were smokers or ex-smokers. The impact of smoking status and COPD on LPS-induced cytokine release by LMs varies markedly from one study to another and from one cytokine to another. Some researchers have reported that LPS-stimulated cytokine production by alveolar macrophages was higher in COPD patients and smokers than in healthy non-smokers (Hodge et al., 2011). However, there were no significant differences in cytokine secretion between current smokers with COPD and non-smokers with COPD (Hodge et al., 2011). In contrast, other studies have found that smoking reduces cytokine production by alveolar macrophages upon stimulation with LPS (Chen et al., 2007) or that current smoking status had no effect (i.e., the dose–response curve for any of the cytokines stimulated by LPS was similar in current smokers and ex-smokers) (Chen et al., 2007; Armstrong et al., 2009, 2011). Furthermore, the inhibitory effect of corticosteroids on the LPS-induced release of cytokines from LMs or alveolar macrophages isolated from bronchoalveolar lavages was similar (i) in non-smokers, current smokers and COPD patients and (ii) after a short (1 h) vs. long (16 h) plate adherence step in the isolation procedure (Armstrong et al., 2011; Plumb et al., 2013; Higham et al., 2014, 2015). Hence, smoking status and COPD impair LPS-induced cytokine release to a variable extent but do not influence the inhibitory effect of corticosteroids on LMs. Therefore, the inhibitory effects of TAS2R agonists observed in the present study are probably not restricted to LMs from ex-smokers or current smokers, and are likely to accurately reflect the *in vivo* responsiveness of human LMs.

In conclusion, we evidenced TAS2R transcript expression in human LMs and identified the TAS2R subtypes that are most likely to be involved in the inhibition of LPS-induced cytokine production. Furthermore, the potential value of TAS2Rs as drug targets for the treatment of chronic obstructive lung diseases is enhanced by the ability of TAS2R agonists to relax airway smooth muscle (Deshpande et al., 2010; Grassin-Delyle et al., 2013), even when β_2_-adrenergic receptors (the current cornerstone target of bronchodilators) are subject to tachyphylaxis (An et al., 2012).

## DATA AVAILABILITY STATEMENT

All datasets generated for this study are included in the manuscript/supplementary files.

## ETHICS STATEMENT

Experiments on human tissue were approved by the regional independent ethics committee board (*Comité de Protection des Personnes Île de France VIII*, Boulogne-Billancourt, France).

## Author Contributions

SG-D and HS conceived the study, performed the experiments, analyzed the data, and critically revised the manuscript. NM helped to draft the manuscript and critically revised it. CA, MB, and EN performed the experiments and analyzed the data. CF and L-JC analyzed the data and critically revised the manuscript. PD managed the study, analyzed the data, performed the statistical analysis, and drafted the manuscript. All authors read and approved the final manuscript.

## Conflict of Interest

The authors declare that the research was conducted in the absence of any commercial or financial relationships that could be construed as a potential conflict of interest.
